# Reconstruction of a human hemicornea through natural scaffolds compatible with the growth of corneal epithelial stem cells and stromal keratocytes

**Published:** 2009-10-17

**Authors:** Vanessa Barbaro, Stefano Ferrari, Adriano Fasolo, Diego Ponzin, Enzo Di Iorio

**Affiliations:** The Veneto Eye Bank Foundation, Venice, Italy

## Abstract

**Purpose:**

To reconstruct a human hemicornea in vitro by means of limbal stem cells cultured onto human keratoplasty lenticules (HKLs) and to obtain a natural corneal graft for clinical applications.

**Methods:**

Limbal stem cells were seeded onto HKLs with or without the presence of feeder layers of lethally irradiated 3T3-J2 cells and compared with the current “gold standard” scaffold, i.e., the fibrin glue. The effects of the scaffold on the preservation of stemness and/or induction of differentiation pathways were investigated through analysis of a variety of markers, including p63 and ΔNp63α for stemness, 14-3-3σ for early differentiation, keratins 3, 14, 12, and 19 to determine cell phenotype,  and α6, β1, and β4 integrins to evaluate interactions with the stroma. Integrity of the stroma was assessed through analysis of keratan sulfate, CD-34 and aldehyde dehydrogenase 3A1 (ALDH3A1) (for keratocytes), visual system homeobox 1 (VSX1), and alpha-smooth muscle actin (α-SMA) (for fibroblasts and myofibroblasts). The structural properties of the reconstructed “hemicornea” were investigated through scanning electron microscopy. To evaluate the preservation of the stemness potential, cells were trypsinized from each scaffold and clonogenic/proliferative characteristics analyzed.

**Results:**

Limbal stem cells expanded onto HKLs gave rise to a stratified squamous keratinized epithelium morphologically similar to that of normal corneas. The resulting corneal epithelium was characterized by basal expression of p63 and ΔNp63α, while expression of 14-3-3σ, keratin 3, and keratin 12 was found in the upper cell layers. The basal cuboidal epithelial cells were anchored to the basement membrane and expressed keratin 14 and α6, β1, and β4 integrins. In the stroma of HKLs, keratocytes maintained the biosynthetic and phenotypic appearances typical of resting/quiescent cells and expressed keratan sulfate, CD-34, and ALDH3A1. Fibroblastic transformation was observed with the appearance of VSX1 and α-SMA. Scanning electron microscopy analysis showed that HKLs maintained their native conformation with collagen fibrils interconnected to the network and parallel to the corneal surface. HKLs did not alter the clonogenic/proliferative capacity of limbal stem cells. No differences were seen when HKL was compared to fibrin glue, one of the scaffolds currently used for limbal stem cell transplantation.

**Conclusions:**

Our findings demonstrate that HKL could be a suitable scaffold for corneal epithelial stem cells as they were shown to proliferate, express differentiation markers, and bind to the underlying stroma with no alterations in clonogenic potential. HKLs have some advantages over currently used scaffolds, such as the possibility to allow cell growth with no feeder layers, to be freeze dried, and to preserve the integrity and viability of stromal keratocytes. The development of a tissue-engineered “hemicornea” might offer new therapeutic perspectives to patients affected by total limbal stem cell deficiency with stromal scarring.

## Introduction

Among the three components of the cornea (epithelium, stroma, and endothelium), only tissue-engineered corneal epithelial cell sheets have been successfully used in clinical applications [1–3]. In vitro cultured corneal endothelial cells have been used to restore corneal transparency in animal models [4] and are ready for clinical studies. In contrast, in vitro reconstructed corneal stroma has never been deemed as clinically feasible, although several attempts have been reported [5]. Both synthetic [6] and natural [7] biological materials are currently used to provide scaffolding support for corneal tissue engineering. Natural materials are more promising because of their physical and mechanical properties [8] and have demonstrated physiologic and biochemical functions equivalent to that of normal corneas. However, all these scaffolds have shown some limitation [9–11].

Depletion/destruction of limbal stem cells causes severe or total limbal stem cell deficiency (LSCD) and results in chronic inflammation, neovascularization, corneal opacity, and eventually visual loss [12]. Potential treatments include transplantation of limbal tissue from the fellow healthy eye either by direct transfer [13] or autologous cultured limbal stem cells (cell therapy) grown onto amniotic membrane (AM) or fibrin gel [14–18]. Despite this, reconstruction of the ocular surface using autologous limbal stem cell transplantation is unable to correct scarring in the Bowman’s layer or stroma of the cornea, which is normally treated as a secondary procedure with penetrating keratoplasty. For this reason, the development of a tissue-engineered product in which autologous corneal stem cells are able to grow onto a scaffold with stroma could provide a potential new treatment for patients with LSCD and stromal scarring. Corneal epithelium has been successfully generated using tissue culture techniques and a variety of scaffolds [19–21]. The AM has been used in several clinical applications of limbal stem cells in patients with LSCD [22,23]. Similarly, fibrin glue was used to treat approximately 200 patients with LSCD, due to chemical or thermal burns, with an overall success rate of 70% [reviewed in 18]. However, none of these scaffolds is devoid of problems. Availability of AM can be an issue, and handling of AM grafts with cultured limbal stem cells not always easy. The fibrin glue is not a porous material, and this might cause stagnation of blood residues underneath the fibrin-cultured stem cells in vivo. More importantly neither AM nor fibrin glue can be considered as a cornea substitute to repair full-thickness stromal scarring. As none of the scaffolds currently available is likely to address this issue, we have decided to evaluate human keratoplasty lenticules (HKLs) [24]. HKLs are currently used for anterior lamellar keratoplasty surgeries and can easily be prepared starting from corneal tissues. They can be used after lyophilization (freeze-dried HKL), a process known to affect keratocyte survival, or immediately after preparation, with the complement of viable keratocytes [24,25].

In this study, we tested the efficiency of HKLs to sustain limbal stem cell propagation with no loss of clonogenic potential. We consider this as a further step toward the development of a tissue-engineered “hemicornea” with corneal epithelial stem cells proliferating and establishing contacts with the keratocytes of the underlying stroma.

## Methods

### Cell cultures

Limbal/corneal keratinocytes were obtained from ocular biopsies taken from whole eye cadaver donors and cultivated as previously described [26,27]. Biopsies were minced and trypsinized (0.05% trypsin/0.01% EDTA) at 37 ºC for 2 h. Once isolated, cells were plated onto lethally irradiated 3T3-J2 cells (2.4×10^4^/cm^2^) and cultured in 5% CO_2_, using a mixture of Dulbecco's Modified Eagle Medium (DMEM) and Ham’s F12 media (2:1) containing Fetal Calf Serum (FCS, 10%; Euroclone, Milan, Italy), penicillin–streptomycin (1%), glutamine (2%), insulin (5 μg/ml), adenine (0.18 mM), hydrocortisone (0.4 μg/ml), cholera toxin (0.1 nM), triiodothyronine (2 nM), and epidermal growth factor (10 ng/ml). Subconfluent primary cultures were passaged at a density of 1.1×10^4^ cells/cm^2^ and cultured as above. Briefly, cells were trypsinized from each scaffold, serially propagated (life span for three passages), and 1,000 of these plated in 100-mm dishes, cultured at low density for 12 days and stained with Rhodamine B [27]. Colony-Forming-Efficiency (CFE) assays (number of colonies generated by seeded cells×100%) and evaluation of the number of cell generations and clonogenic cells were used to assess the presence of epithelial stem cells.

### Human keratoplasty lenticules

HKLs were prepared as previously described [24]. Briefly, corneoscleral rims were preserved at 4 °C and used within 1–2 days from their excision. Each specimen was firmly placed in an artificial chamber and the epithelium manually removed after bathing the surface of the cornea with an isotonic solution containing 5 mg/ml of trypsin and 2 mg/ml of EDTA (Sigma-Aldrich, Milan, Italy). HKLs were obtained by microkeratome resection of epithelium-free corneas.

### Preparation of hemicorneas in vitro

Limbal/corneal keratinocytes were plated onto HKLs, with or without feeder layers of lethally irradiated 3T3-J2 cells, at a concentration of 5×10^4^ cells/cm^2^. Hemicorneas were cultured under submerged conditions for 7 days and air lifted for 14 further days. Corneal epithelial cells were also plated on fibrin sealant, in the presence of 3T3-J2 feeder layers, and cultivated at the air–liquid interface for 14 days.

### Histology

Hemicorneas were fixed in 4% paraformaldehyde (overnight at 4 °C), embedded in optimal cutting temperature (OCT) compound, frozen, and sectioned. Sections (5–7 μm) were analyzed by indirect immunofluorescence.

### Immunofluorescence

Immunofluorescence studies were performed by using antibodies against pan-p63 (4A4, mouse monoclonal, 1:100, BD Biosciences, Milan, Italy), p63ΔNα (rabbit polyclonal, 1:200; PRIMM, Milan, Italy), keratin 12 (K12, sc-17099, goat polyclonal, 1:100; Santa Cruz Biotechnology, Santa Cruz, CA), keratin 3 (K3, AE5 clone, mouse monoclonal, 1:100; MP Biomedicals, Solon, OH), keratin 19 (K19, RB-9021, rabbit polyclonal, 1:200; NeoMarkers, Freemont, CA), 14-3-3σ (rabbit polyclonal, 1:400; PRIMM), keratin 14 (K14, ep1612y, rabbit monoclonal, 1:100; Epitomics, San Bruno, CA), integrin β1 (mab1965, mouse monoclonal, 1:100; Chemicon International, Padova, Italy), integrin α6 (mab1358, mouse monoclonal, 1:100; Chemicon International), integrin β4 (CD104, mca1456f, mouse monoclonal, 1:100; Serotec, Milan, Italy), VSX1 (sc-22547, goat polyclonal, 1:50; Santa Cruz Biotechnology), α-SMA (mouse monoclonal, 1:100; Sigma-Aldrich), CD34 (H-140, sc-9095, rabbit polyclonal, 1:100; Santa Cruz Biotechnology), keratan sulfate (KS, 5-D-4, mouse monoclonal, 1:200; Seikagaku Corporation, Tokyo, Japan), and aldehyde dehydrogenase 3A1 (ALDH3A1, sc-137168, mouse monoclonal, 1:200; Santa Cruz Biotechnology) for 1 h at 37 °C. Rhodamine and fluoroscein isothiocyanate-conjugated secondary antibodies (1:100; Santa Cruz Biotechnology) were incubated for 1 h at room temperature. Specimens were analyzed with an LSM 510 Meta Confocal Microscope (Zeiss SpA, Milan, Italy). Image analysis was performed using the LSM 510 software (Zeiss SpA, Milan, Italy).

### Scanning electron microscopy

For electron microscopy studies (JSM-6490; Jeol, Welwyn Garden City, UK), cryosections from the reconstructed hemicorneas were fixed with 2% glutaraldehyde in 0.1 M PBS (pH 7.2) at 37 °C for 1 h, washed three times in buffer for 10 min, fixed in 1% osmium tetroxide for 1 h at 37 °C, and dehydrated through a graded series of ethanol. All specimens were critical point dried (EM CPD030; Leica Microsystems, Wetzlar, Germany), coated with gold using a Sputter Coater (S150A; Edward, UK), and observed at magnifications between 100× and 3,500×.

## Results

### Epithelium and stroma of reconstructed human keratoplasty lenticules resemble those of normal corneas

#### The epithelium

**Figure 1:**
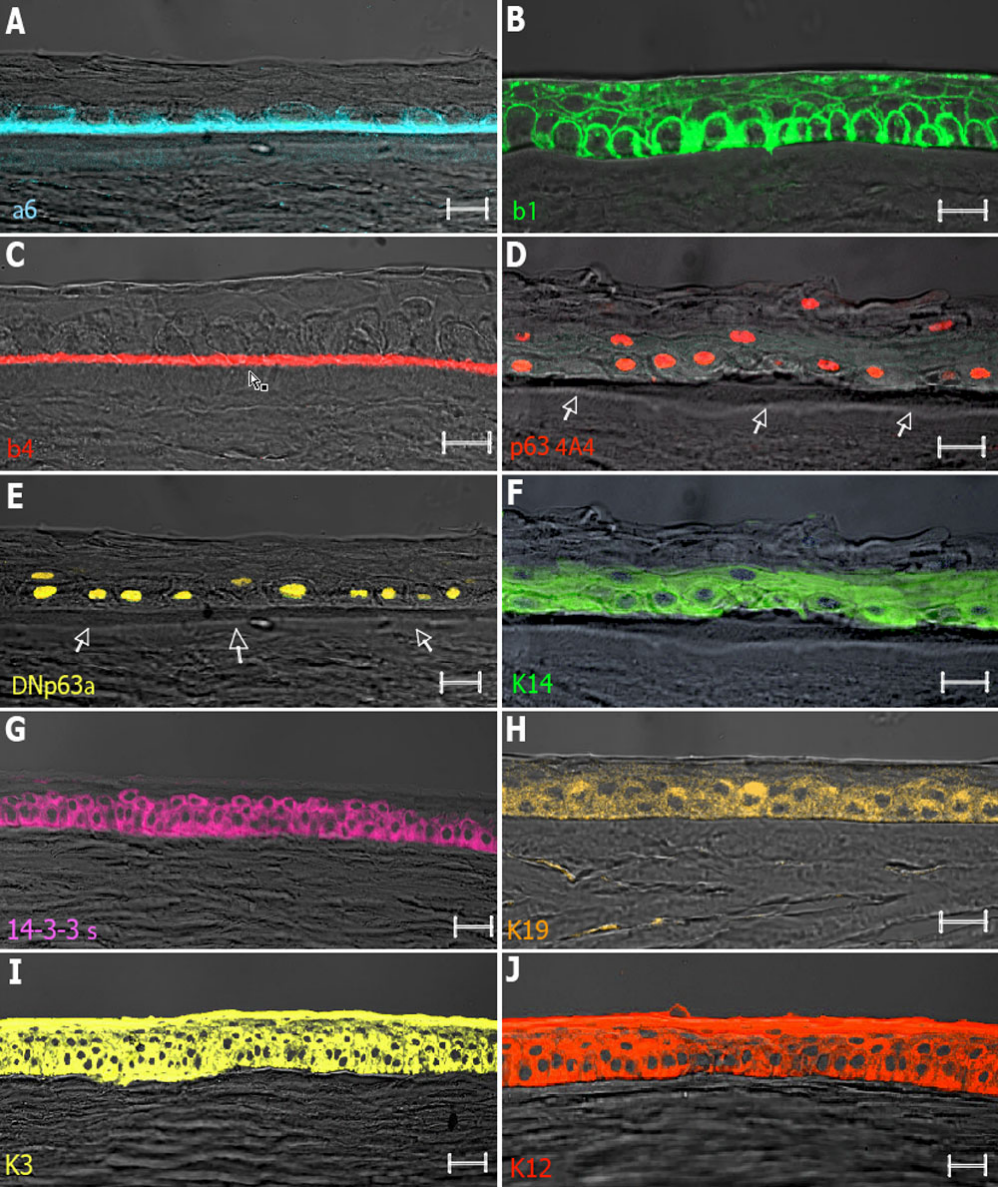
Expression of epithelial cell markers in reconstructed hemicorneas. Human corneal epithelial stem cells seeded onto the HKL scaffold form a pluristratified and differentiated epithelium. Cryosections were analyzed through immunofluorescence. The layer of basal cuboidal cells was firmly attached to the underlying extra cellular matrix (ECM) and to the basement membrane through integrins α6 (blue, **A**), β1 (green**, B**), and β4 (red, **C**). The basal expression of p63 isoforms (red, **D**) and of the stem cell marker ΔNp63α (yellow, **E**) was always observed, thus suggesting the maintenance of undifferentiated progenitor cells interspersed between differentiated cells. Expression of keratin 14 (green, **F**) was also observed in the basal layers. Corneal differentiation occurred in all epithelial layers and was evaluated through the analysis of several markers, including 14-3-3σ (pink, **G**), keratin 19 (orange, **H**), keratin 3 (yellow, **I**), and keratin 12 (red, **J**). Note the presence of the Bowman’s membrane in transmitted-light images (white arrows, **D**–**E**). Scale bar=50 μm.

Human keratoplasty lenticules (HKLs) are naturally derived matrices obtained from human corneas [24] that we evaluated for their ability to mimic the local environment of human corneal epithelial stem cells and provide a template for cell growth and extra cellular matrix (ECM) production. Primary human corneal epithelial stem cells were seeded onto HKLs and allowed to grow for 7 days. As shown in Figure 1, the resulting epithelium was well organized and stratified into four to five cell layers, with basal cuboidal cells differentiating upward to winged cells. The layer of basal cuboidal cells was firmly attached to the underlying ECM and to the basement membrane (Figure 1A-C) through integrins α6, β1, and β4.

Maintenance of stemness potential and differentiation pathways are both important factors for the development of healthy corneal epithelia. For this reason, specific markers of stemness and differentiation were evaluated. Basal epithelial cells expressed p63, the epithelial stem cell marker ΔNp63α and K14 (Figure 1D–F). Differentiation pathways were not altered, as indicated by the expression of 14-3-3σ (Figure 1G), an early differentiation marker for stratified epithelia [28], and of different specific cytokeratins of the ocular surface, such as keratin 19, keratin 3, and keratin 12 (Figure 1H–J). We also observed the presence of a basement membrane underneath the epithelial cell layers and preservation of the Bowman’s membrane (see arrows in Figure 1D,E). Importantly, expression of the different markers resembled that observed in normal corneal epithelia (data not shown), thus suggesting that HKLs are able to support the growth and maintain the differentiation pathways of corneal epithelial stem cells.

**Figure 2:**
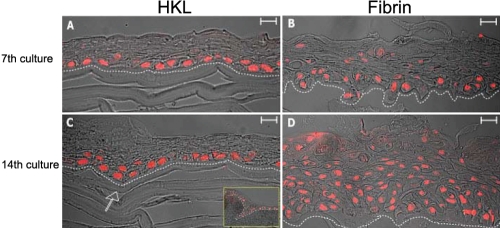
Comparison between HKL and fibrin-glue matrix as scaffolds for cultured human keratinocytes. Human corneal epithelial stem cells were cultured onto HKL (**A**, **C**) and fibrin glue (**B**, **D**) in submerged conditions for 7 days (**A**, **B**) and at the air–liquid interface for 14 further days (**C**, **D**). Increased stratification (more than 15 cell layers) and overexpression of p63 isoforms in suprabasal cells were only observed when the fibrin glue was analyzed (**D**). The growth onto HKL showed a phenotype that was more similar to that of normal human corneal epithelia (**A**, **C**). Note that the basal epithelial plane became undulated, yielding an appearance that resembles the palisade of Vogt (bottom inset, **C**). Scale bar=50 μm.

It is known and has been previously reported [29] that keratinocytes cultured at the air–liquid interface give rise to a multilayered epithelium, thus mimicking the naturally occurring conditions of corneal epithelia. Human corneal epithelial cells seeded onto HKLs were therefore cultured in submerged conditions for 7 days (Figure 2A) and, once confluent, air lifted at the air–liquid interface for 14 further days (Figure 2C). The epithelium appeared morphologically similar to that of a normal human cornea (data not shown). The basal epithelial plane became undulated, yielding an appearance that resembles the digital invasion of the limbal basal epithelium in the palisade of Vogt (Figure 2C). This morphologic feature became more evident on day 14.

In all these experiments, fibrin glue was used as the “gold-standard” scaffold, as previously seen to improve the engraftment of epithelial stem cells in patients with full-thickness burns or LSCD [14] without interfering with the normal differentiation pathways. Increased stratification and hyperproliferation were only observed when fibrin glue was used, but not with HKLs, and associated with overexpression of p63 in suprabasal cells (Figure 2B,D). The number of epithelial cell layers onto the fibrin increased dramatically from day 7 to day 14, with some areas having more than 15 cell layers. No difference in the number of cell layers was observed in HKLs, either at 7 or 14 days. A likely explanation for this difference could be that while fibrin is an inert scaffold supporting keratinocyte growth but unable to generate specific signals regulating cell proliferation, stratification, and spatial organization, HKL is an active matrix able to affect, through cell-to-cell communication, all the aspects of keratinocyte growth, including the number of cell layers.

#### The stroma

**Figure 3:**
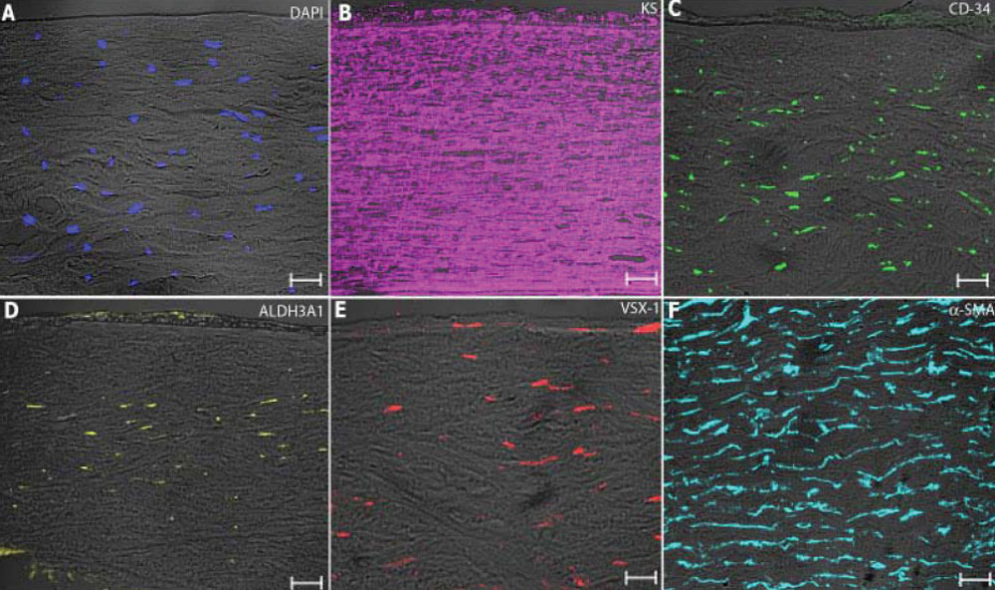
Keratocytes in the stromal part of the hemicornea were observed after 4',6-diamidino-2-phenylindole (DAPI) staining (**A**). Expression of specific markers of the corneal stroma was analyzed: keratan sulfate (KS), essential for the maintenance of the correct orientation of collagen fibrils (**B**); CD-34, a specific marker of keratocytes expressed both in vivo and in vitro (**C**); ALDH3A1, a marker of quiescent keratocytes in vivo (**D**); VSX1, found in keratocytes undergoing fibroblastic transformation (**E**); α-SMA, a muscle protein of F-actin stress fibers, typically found in myofibroblasts (**F**). Scale bar=50 μm.

When the stroma of the reconstructed hemicorneas (i.e., HKLs with corneal epithelial cells) was analyzed, the expression pattern of specific markers was found to be similar to that of wounded human corneas (data not shown). This was highly expected as the lenticules used in our study were at least a few days old (time required by the eye bank personnel to assess unsuitability for transplantation plus days in culture), a period of time likely to have induced activation of wound-healing responses/pathways [27]. 4',6-diamidino-2-phenylindole (DAPI)-stained keratocytes (Figure 3A) were surrounded by abundant ECM and expressed keratan-sulfate proteoglycans (Figure 3B). The latter are essential components of the corneal stroma as they are required for the maintenance of the correct orientation of collagen fibrils and therefore crucial for preserving corneal transparency. Keratocytes appeared quiescent, flattened, and expressed both CD-34 (Figure 3C), a glycosylated transmembrane protein normally expressed in vivo and in vitro [27], and aldehyde dehydrogenase 3A1 (ALDH3A1), a marker of quiescent keratocytes in vivo, shown to be downregulated during wound healing and essential for the maintenance of a stable and transparent corneal structure [30] (Figure 3D). As expected, we also found keratocytes undergoing fibroblastic transformation, as they expressed VSX1 (Figure 3E), developed F-actin stress fibers containing α-SMA (Figure 3F) protein, and exhibited a myofibroblastic phenotype (Figure 3F).

### Scanning electron microscopy-based studies do not show structural differences between human keratoplasty lenticules and normal corneas

**Figure 4:**
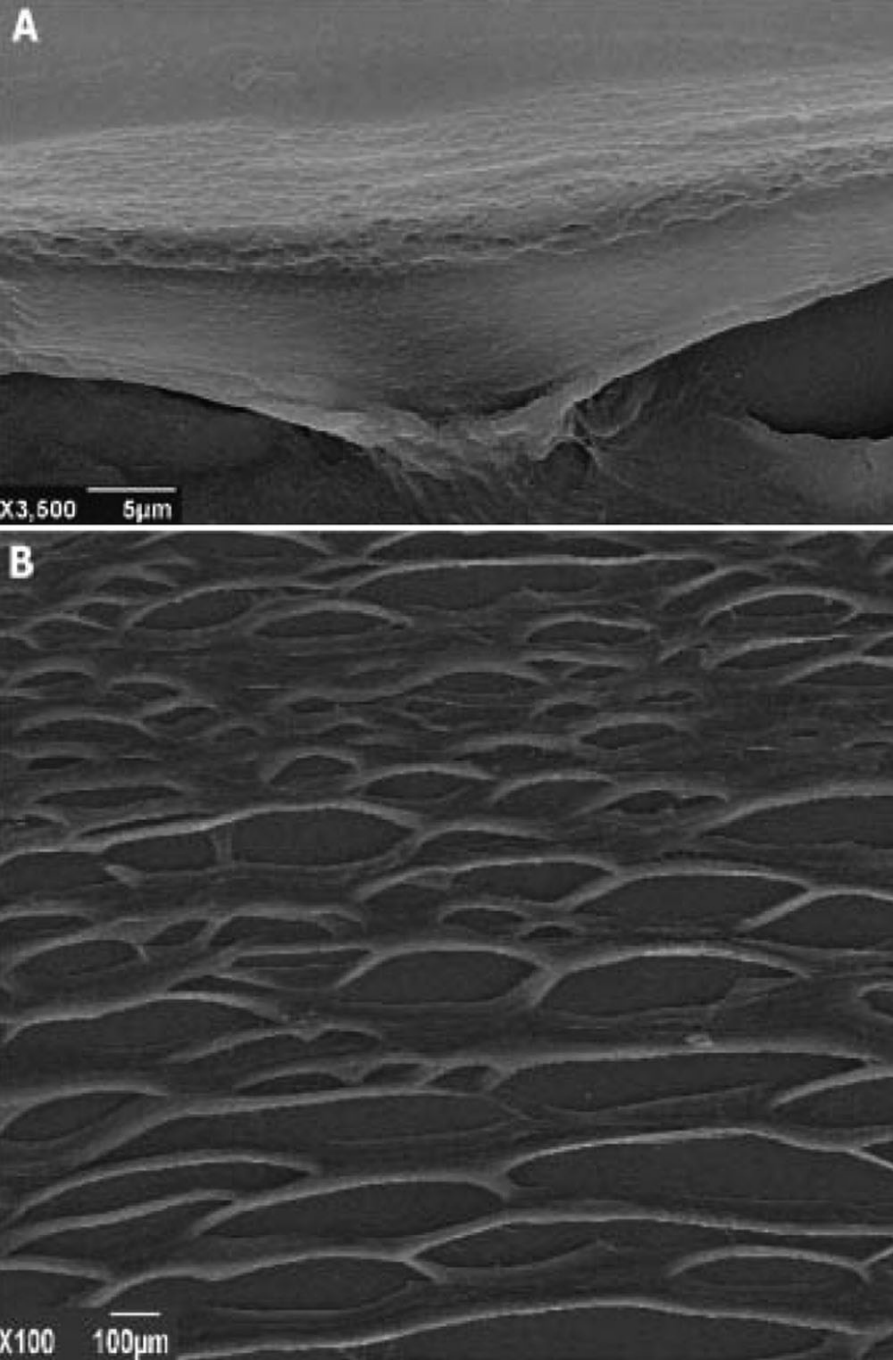
Transparency and biomechanical properties of the cornea depend on the structure and organization of corneal stroma. Scanning electron microscope (SEM) images of the HKL showed that the Bowman's membrane structure maintained its native conformation (**A:** 3500×, scale bar 5μm). Collagen fibers and fibers interconnecting to the network formed collagen bundles, which were regular and parallel to the corneal surface (**B**: 100×, scale bar 100 μm). These were similar to those observed in normal human corneas.

Transparency and biomechanical properties of the cornea depend on the structure and organization of the corneal stroma. Knowledge of these properties is, therefore, important for the development of an adequate model of tissue-engineered cornea. As shown in Figure 4A,B, scanning electron microscopy-based studies of the grafts comprising HKLs and limbal stem cells did not show any change or alteration in fibril organization and Bowman's membrane structure compared to normal human corneas (data not shown).

### Human keratoplasty lenticules do not alter the clonogenic ability and proliferative potential of limbal stem cells

**Figure 5:**
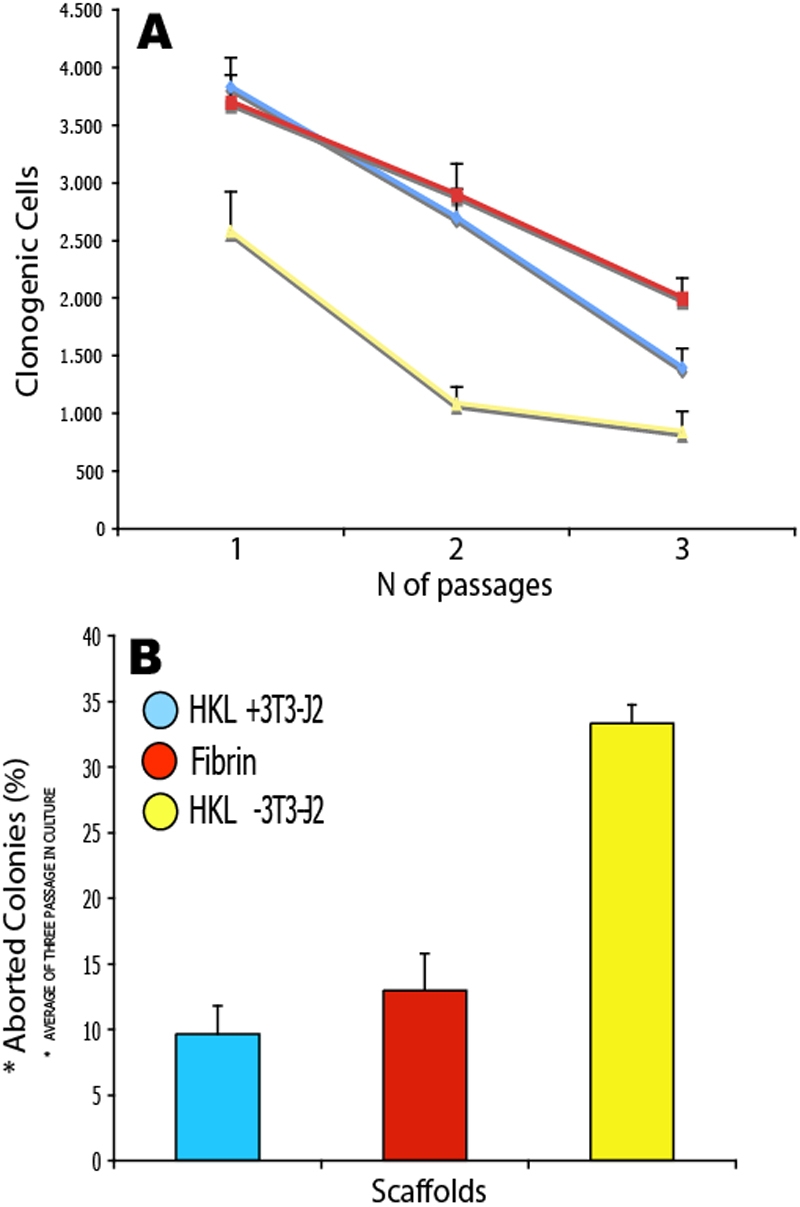
Cells isolated from each of three scaffolds were serially propagated for three passages. This allowed us to obtain information about the residual clonogenic potential of the cells grown onto the scaffolds and to evaluate the effects of the matrix on the preservation of stemness and induction of differentiation pathways. [HKL + 3T3/J2] = HKLs with 3T3-J2 feeder layer; [HKL - 3T3/J2] = HKLs without 3T3-J2 feeder layer. No difference in the number of clonogenic cells (**A**) or percentage of aborted colonies (aborted colonies/total colonies ratio; **B**) was observed when cells isolated from HKLs (in the presence of 3T3-J2 feeder layers) or fibrin were compared. In the absence of a 3T3-J2 feeder layer, reduced number of clonogenic cells and increased percentages of aborted colonies were observed. Despite this, cells were found proliferating for at least three passages in culture, thus suggesting that HKLs might not interfere with the stemness and proliferative potential of corneal stem cells. Error bars indicate SEM (n=3).

In regenerative medicine, the maintenance of stem cells in culture is of crucial importance. Any new scaffold should, therefore, not alter the clonogenic and proliferative potential of stem cells. In order to obtain information about the residual clonogenic potential of the cells grown onto the scaffolds and evaluate the effects of the matrix on the preservation of stemness and induction of differentiation pathways, cells were trypsinized from the scaffolds, serially propagated, and analyzed by means of cell-biology parameters (Figure 5). No differences in the number of clonogenic cells (Figure 5A) or in the percentage of aborted colonies (Figure 5B) were observed when we compared cells isolated from HKLs (in the presence of 3T3-J2 feeder layers) or fibrin.

We also tested whether it was possible to culture human limbal stem cells without using feeder layers of murine origin (Figure 5). This would avoid or at least reduce potential immune responses due to the presence of xenogenic material whenever HKLs will be used for clinical applications. As shown in Figure 5, HKLs with no 3T3-J2 feeder layers showed a loss in clonogenic potential and increased percentages of abortive colonies, more evident from the second passage onward.

## Discussion

The replacement of diseased tissues and organs by means of tissue engineering approaches is rapidly becoming a reality and the possibility to obtain bioengineered corneal replacements recently demonstrated [6–10,17–19]. Successful tissue engineering depends on the availability of suitable scaffolds during the initial stages of reconstruction [31,32]. The choice of an appropriate matrix is, therefore, of crucial importance. The ideal scaffold should fulfill several requirements, including biocompatibility and the possibility to be repopulated with autologous recipient cells. Various types of matrices, such as polymers, amniotic membrane, or fibrin gel, have been investigated for ocular surface reconstruction [14,19–21]. However, none of them is completely devoid of problems. Compared to these matrices, HKLs appear to be particularly attractive because of their anatomic similarity to the human cornea, with the stroma acting as a physiologically functional tissue substitute and not simply as a scaffold for limbal stem cell propagation. Our model might, therefore, be considered as a step forward toward the development of a tissue-engineered hemicornea in which corneal epithelial stem cells (i) proliferate, (ii) establish contacts with the keratocytes of the underlying stroma, and (iii) renew the epithelium by producing transiently amplifying cells and differentiated cell layers. Keratin 3 and 12, the specific corneal epithelial differentiation markers, are expressed from the second layer of the epithelium to the winged cells. Interaction with the basement membrane was demonstrated by the continuous expression of several integrins. This is an important feature of our reconstructed epithelium as integrins have been shown to lead to cell signaling cascades controlling diverse processes, such as migration, proliferation, cell survival, and cellular phenotype [33].

The possibility of cultivating limbal epithelial stem cells onto HKLs in vitro and of obtaining a physiologically functional tissue with proliferating keratinocytes and keratocytes are important features that make HKL a potential and interesting scaffold for the treatment of LSCD. In addition, HKLs have some further advantages that are worth discussing in depth.

Firstly, the use of HKL could overcome some of the problems associated with the use of fibrin gel and improve fibrin-cultured limbal stem cell transplantation. Occasionally, in fact, corneal stem cell proliferation causes a progressive thinning of the fibrin glue, thus making manipulation of stem cell grafts by surgeons extremely difficult. In addition, as the fibrin glue is not a porous material, this might cause stagnation of blood residues underneath the fibrin-cultured stem cells in vivo, thus not allowing (i) optimal reabsorption of the fibrin and (ii) interaction of keratinocytes with the underlying corneal stroma.

Another potential advantage of HKLs is their ability to sustain corneal stem cell proliferation even in the absence of lethally irradiated 3T3-J2 feeder layers. Murine feeder layers are essential for the maintenance of the stemness and proliferative potential of epithelial stem cells cultured in vitro [14]. However the development of feeder-free culture conditions for corneal epithelial stem cells would be of great interest. In fact, although lethally irradiated and therefore unable to replicate, murine 3T3-J2 fibroblasts are still present in grafts suitable for transplantation with percentages of approximately 5% (data not shown). Limbal stem cell transplantation, as it is currently carried out, might therefore induce inflammatory responses against the xenogeneic component of the graft (the murine fibroblasts), thus potentially reducing the possibilities of successful outcomes. In addition, it is very likely that, in the near future, guidelines regulating the clinical applications of stem cells will require the development of animal-free culture systems (no murine feeder layers or culturing media with animal-derived proteins/growth factors). HKL could therefore be an advantageous scaffold for limbal stem cells. In fact, while we observed a reduced number of clonogenic cells and increased percentages of aborted colonies, cells were able to proliferate for at least three passages in culture, thus suggesting that HKLs might not interfere with the stemness and proliferative potential of corneal stem cells. Further studies are, however, necessary to determine the ability of HKLs to support the maintenance of holoclones in cultures rather than transient amplifying cells only.

A third interesting feature of HKLs is the possibility to be freeze dried, thus eliminating viable, and potentially immunogenic, keratocytes. Recently, various decellularization procedures have been used to eliminate cells and create a cell-free matrix [19,20,34], which can be repopulated with recipient cells after implantation [25,35]. Advantages would not only be limited to the lower antigenicity of freeze-dried HKLs. Stability, safety, and sterility are all properties that would make HKLs more suitable than other carriers in the strictly regulated Good Manufacturing Practice (GMP) settings that are now required for production/manufacture of cell-therapy products for human use [36].

In the future, the possibility of culturing and expanding limbal stem cells onto HKLs might open up new and intriguing perspectives for the surgical treatment of LSCD. The management of LSCD is currently carried out using a multistaged approach. The ocular surface is firstly stabilized through pannus resection and transplantation of autologous cultured limbal stem cells. Visual rehabilitation is normally gained through a second stage involving penetrating keratoplasty. Improvements in microsurgical techniques and introduction of new devices have recently led to increasing numbers of lamellar keratoplasty procedures being performed. This appears to be true both for the anterior lamellar keratoplasty (ALK), replacing the anterior stroma, and for the posterior lamellar keratoplasty (PLK), which involves the replacement of deep stromal and endothelial layers. Lenticules for both ALK and PLK are prepared by surgeons just before surgery or, very often, provided by eye banks. In cases of LSCD patients with eyes having milder and superficial stromal scarring, transplantation of autologous limbal stem cells cultured onto HKLs might, therefore, be performed using ALK surgical procedures. In these cases the multistaged approach described earlier would be replaced by just one step in which grafts of HKL with limbal stem cells would replace the damaged stroma of the recipient at once. This technique would also provide tectonic tissue support to LSCD corneas, which are normally thinner and have higher risks of perforations than healthy ones.

Finally, the use of HKLs could provide an interesting in vitro organotypic culture system for:

(i) The evaluation of the growth, proliferation, and differentiation processes of corneal stem cells from patients with disorders/pathologies that make propagation of cells onto commonly used plastic Petri dishes difficult.

(ii) The development of new pharmaceutical drugs (e.g., eye drops, medicinal products), as they might represent a valid in vitro-based alternative method for assessing toxicity [37] and safety.

Future studies will need (i) to test the efficiency of hemicorneas in clinical applications for the treatment of LSCD and (ii) to obtain a complete corneal equivalent by plating limbal/corneal keratinocytes onto lenticules normally used for PLK, i.e., complete with Descemet’s membrane and endothelium.
